# Identifying Key Factors Associated with the Spatiotemporal Dynamics of Fish Assemblages in Subtropical River-Type Reservoir Ecosystems: Implications for Fishery Management and Conservation

**DOI:** 10.3390/ani16182892

**Published:** 2026-09-14

**Authors:** Chengjie Yin, Rongzhen Zhang, Zewen Feng, Xiaoning Shi, Renhui Li, Miao Xiang, He Zhang, Longgen Guo

**Affiliations:** 1 School of Resources and Environmental Engineering, Suzhou University, Suzhou 234000, China; ychj100@163.com; 2 National Engineering Laboratory for Lake Pollution Control and Ecological Restoration, Institute of Hydrobiology, Chinese Academy of Sciences, Wuhan 430072, China; 3 Wenzhou Shanxi Hydro-Junction Management Center, Wenzhou 325035, China; rongzhen_zh@163.com (R.Z.); wzwcfzw@163.com (Z.F.); 4 Taishun Ecological Environmental Monitoring Station, Wenzhou Bureau of Ecology and Environment Taishun Branch, Wenzhou 325500, China; noranxs@163.com; 5 National and Local Joint Engineering Research Center of Ecological Treatment Technology for Urban Water Pollution, Zhejiang Provincial Key Laboratory for Water Environment and Marine Biological Resources Protection, College of Life and Environmental Sciences, Wenzhou University, Wenzhou 325035, China; renhui.li@wzu.edu.cn; 6 Yangtze River Fisheries Research Institute, Chinese Academy of Fishery Sciences, Wuhan 430223, China; xiangmiaoihb@163.com

**Keywords:** river-type reservoirs, fish community, spatial distribution, environmental variables, fisheries management

## Abstract

Characterizing the spatiotemporal patterns of fish communities in subtropical river-type reservoirs provides useful baseline information for fisheries stewardship and aquatic ecosystem monitoring. This work investigated fish assemblages across 18 sampling sites within Shanxi Reservoir, Zhejiang Province, using an integrative approach combining gillnet surveys, hydroacoustic detection, and habitat parameter monitoring. Sampling was conducted during two periods: October 2022 (autumn, post-flood, fattening phase) and April 2023 (spring, normal water level, spawning phase). A total of 22 species were documented in spring and 21 in autumn, with Cyprinidae species predominating. Dominant species shifted seasonally, led by *Hypophthalmichthys nobilis*, *Culter dabryi* and *Hemiculter leucisculus*. Planktivores and carnivores were more abundant in spring, while omnivores increased in autumn. Spatially, fish populations were mainly concentrated in the shallow riverine zone (SRZ), predominantly comprising small- to medium-sized individuals. Mantel tests and Pearson correlation analyses identified associations between fish assemblage structure in the deep lake zone (DLZ) and environmental variables including dissolved oxygen, conductivity, chlorophyll a, and phytoplankton density and biomass. This study provides preliminary baseline data on fish community dynamics in Shanxi Reservoir and offers tentative observations that may help inform future zoned monitoring and ecological restoration planning.

## Simple Summary

As an exploratory two-season baseline survey, this study characterized fish communities in Shanxi Reservoir, a subtropical river-type reservoir in Zhejiang Province, during spring and autumn. A total of 27 species were recorded, with clear seasonal succession in dominant species. Planktivorous and carnivorous fish were more abundant in spring, while omnivorous fish increased in autumn. Fish were mainly concentrated in the shallow riverine zone, and community composition was associated with environmental factors such as dissolved oxygen and chlorophyll a. These preliminary observations provide baseline data for future long-term monitoring and may inform tentative fisheries management strategies.

## 1. Introduction

Reservoirs constitute a cornerstone of water supply security and function as the primary source of drinking water for numerous densely populated regions [1,2]. Among reservoir types, river-type reservoirs are distinguished by pronounced longitudinal riverine attributes, chiefly reflected in pronounced seasonal oscillations of discharge and water level, alongside spatially heterogeneous habitats such as vertically stratified prey fields [3,4]. Within these systems, fish assemblages occupy a central trophic position, playing a pivotal role in mediating nutrient cycling and energy transfer throughout the aquatic food web. Understanding the structural composition and spatiotemporal dynamics of these assemblages holds significant theoretical and practical value, being crucial for constructing food web construction, ecological process analysis, and fisheries management [5,6,7]. Notably, unlike the relatively stable conditions of lake-type reservoirs, river-type reservoirs preserve inherent fluvial properties that collectively govern the spatial distribution and seasonal variability of fish communities [8,9,10,11,12,13,14,15,16].

Seasonal and spatial dynamics of reservoir fish communities are driven by a complex interplay of hydrological and anthropogenic factors, such as temperature fluctuations, water level changes, nutrient inputs, food availability, and habitat heterogeneity [17,18,19,20]. For example, temperature variability directly influences fish physiology by modulating metabolic rates, feeding capacity, and reproductive timing through its effect on body temperature [21]; concurrently, it restructures prey availability by altering plankton community composition and distribution [22]. Monsoon-driven hydrological pulses intensify tributary inflows, delivering nutrients that generate distinct habitat structure characterized by nutrient-rich tributary zones versus deep lake zones with limited prey, thereby triggering cascading changes in fish community composition and spatial distribution [23,24,25,26,27,28,29,30]. Furthermore, seasonal water level fluctuations dynamically reshape littoral habitats, inundating or exposing spawning grounds and foraging areas, which in turn strongly influence the seasonal assembly and spatial partitioning of fish assemblages [31,32]. Identifying environmental drivers underlying fish distribution is therefore critical both for elucidating ecosystem functioning and for informing adaptive, evidence-based reservoir management.

Shanxi Reservoir (SXR) is situated in the subtropical Feiyun River basin of Zhejiang Province, serving as a large river-type reservoir primarily designed for water supply, while also serving critical roles in flood control and fisheries. Stretching approximately 30 km in length and reaching a maximum width of 3 km [2,33], SXR functions as a key aquaculture and fishery hub, supporting over 30 fish species including economically significant ones like *Hypophthalmichthys molitrix*, *H. nobilis*, *Culter* spp. (*Culter alburnus*, *Culter mongolicus*, *C. dabryi*) and *Coptodon zillii* (CJ. Yin Unpublished data). In recent years, however, upstream water pollution has increasingly threatened the reservoir’s ecological integrity. Recurrent eutrophication such as excessive algal proliferation, has been observed in several bays, leading to seasonal deterioration of water quality and posing risks to drinking water safety [34]. Studies indicate that strategic stocking of filter-feeding fish can effectively suppress algal blooms and improve water clarity [35]. As environmental pressures mount, sustainable reservoir fisheries management must harness the ecological services provided by stocked fish, yet this requires a robust understanding of local ecological conditions and fish community structure [36]. Current research on SXR has largely centered on water quality assessment, macroinvertebrate community dynamics, phytoplankton and zooplankton ecology. Notably absent are comprehensive studies on the fish community, its composition, spatiotemporal dynamics, trophic interactions, and functional roles. This knowledge gap impedes effective water quality regulation, hinders the development of ecologically informed fisheries practices, and limits our capacity to formulate science-based, adaptive management strategies for the reservoir [37,38,39,40].

Accordingly, this study systematically investigated the water quality, phytoplankton, and fish communities in the SXR. We hypothesize that: (H1) Temporal shifts in plankton density and biomass regulate the structure and composition of fish communities; (H2) The SRZ and DLZ support distinct fish assemblages, specifically, the SRZ exhibits higher species richness and abundance, whereas the DLZ sustains relatively lower diversity and density; (H3) Trophic guild composition varies seasonally: planktivorous and herbivorous fishes dominate in summer, while piscivores increase in relative dominance during spring, likely due to seasonal aggregation of prey in thermally stable, deeper habitats. The study therefore aimed to: (1) Characterize the seasonal and spatial dynamics of fish community structure across SXR; (2) Test the above hypotheses by quantifying the relative influence of trophic resource availability (e.g., plankton biomass, habitat stability) on fish assemblage patterns; and (3) determine whether the SRZ and DLZ harbor functionally and structurally distinct assemblages. This research not only fills a gap in SXR fish and fishery studies but also provides exploratory baseline research for water quality management, ecological restoration, and scientific fishery management in this and other similar reservoirs.

## 2. Materials and Methods

### 2.1. Study Area

**Figure 1:**
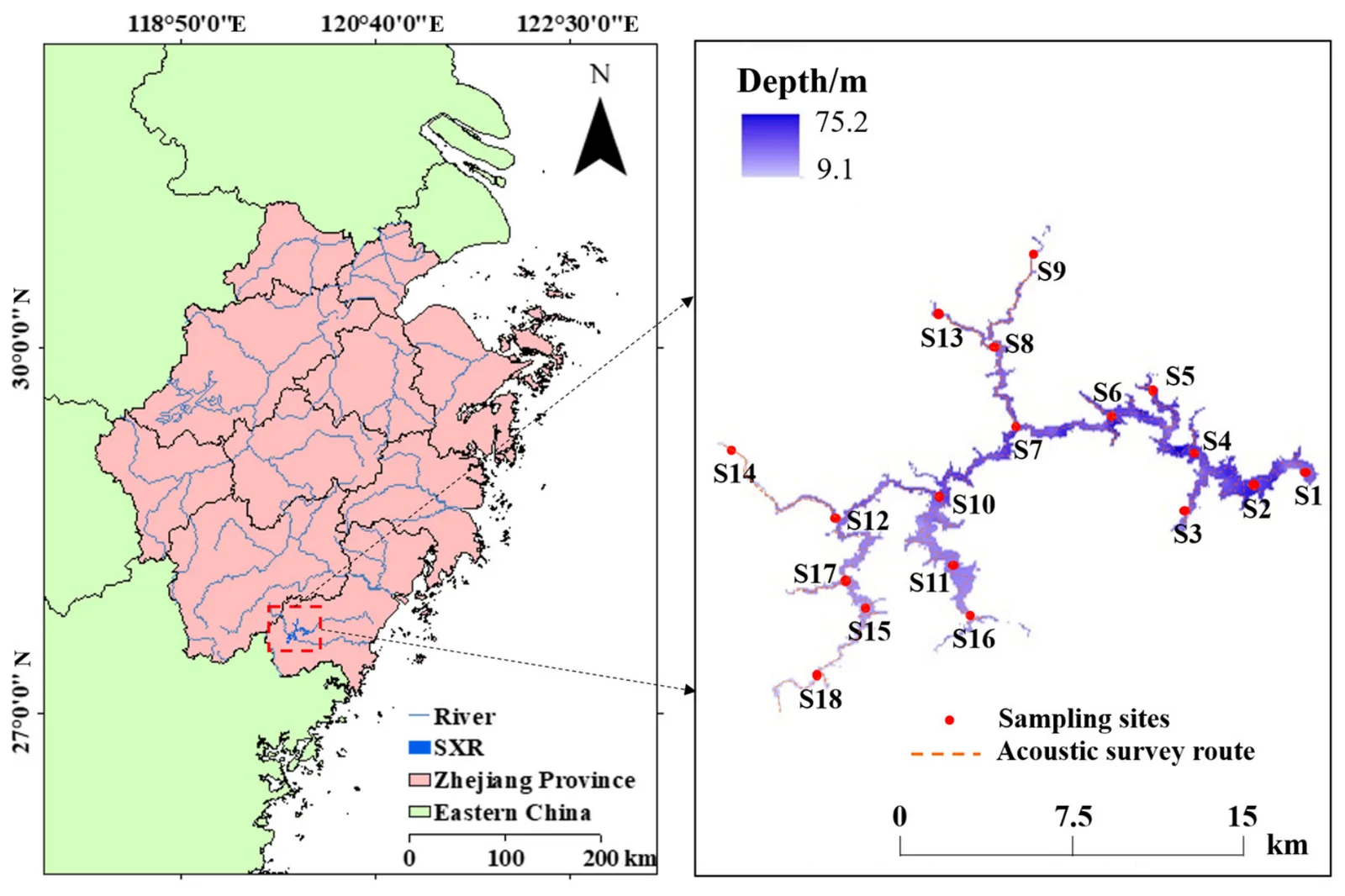
Geographic location of Shanxi Reservoir (SXR) in the Feiyun River basin, Zhejiang Province, China, and spatial distribution of the 18 sampling sites across the shallow riverine zone (SRZ) and deep lake zone (DLZ) used in this two-season baseline survey.

SXR (27°36′–27°50′ N, 119°47′–120°15′ E), located in Wenzhou City, Zhejiang Province, serves as a vital drinking water source for the city and is designated as one of China’s nationally important drinking water reservoirs (Figure 1). It drains a catchment area of 1529 km^2^ and has a total storage capacity of 1.82 × 10^9^ m^3^, receiving inflow from 14 major tributaries including Huangtankeng [36,37]. As a representative deep-valley reservoir in southeastern China, SXR experiences a subtropical humid monsoon climate, with an average annual temperature of 18 °C and mean annual precipitation of 1870 mm.

Field sampling was conducted during two ecologically and hydrologically significant periods: October 2022 (autumn—post-flood season, corresponding to the biota’s fattening phase) and April 2023 (spring—at normal water level, coinciding with the spawning phase of resident aquatic organisms). These timepoints were carefully selected to align with both the reservoir’s hydrological regime and the reproductive phenology of its native biota. Following established protocols [41] and the reservoir’s actual ecological gradients, the study area was partitioned into two distinct ecological zones: the deep lake zone (DLZ), comprising nine stations (S1–S7, S10, S12), all situated in water deeper than 40 m; the shallow riverine zone (SRZ), comprising nine stations (S8, S9, S11, S13–S18), all shallower than 40 m.

Ecologically, the SRZ typically receives higher nutrient inputs from inflowing tributaries, experiences stronger hydrological pulses, and exhibits greater habitat heterogeneity (e.g., shallow littoral areas, submerged macrophytes) [3]. These conditions favor planktivorous and omnivorous species with flexible feeding strategies. In contrast, the DLZ is characterized by deeper water columns, pronounced thermal stratification, lower nutrient concentrations, and reduced prey diversity, which often support carnivorous taxa and species adapted to more stable, resource-limited environments [31]. Recognizing these distinct ecological mechanisms is essential for zone-specific management: stocking filter-feeding fish in the SRZ may more effectively control phytoplankton blooms [35], whereas monitoring dissolved oxygen and prey availability in the DLZ can help anticipate shifts in carnivorous fish populations.

### 2.2. Sample Collection and Analysis

This study employed comprehensive catch surveys at each sampling site through field sampling using multiple gear combinations. All fish sampling was conducted as part of official, routine fishery resource monitoring programs mandated under the Fisheries Law of the People’s Republic of China and following the national industry standard Specifications for Freshwater Fishery Resource Surveys (SC/T 9455-2025) [42]. Specifically, at each site, three bottom-set gillnets (30 × 0.45 m, mesh 5 mm) were deployed. Additionally, composite gillnets (50 m long, 3 m high, mesh 3.5 mm) featuring a dual-layer buoyant-sinking structure were deployed. The gear combination at each site also included a set of three-layer gillnets, each measuring 100 m in length and 20 m in height, with mesh sizes of 40, 80, 120, and 160 mm, comprising one bottom-set and one floating net. Nets were set at 18:00 and retrieved at 06:00, operating for 12 h. Upon retrieval, fish were identified, measured, and counted while still alive [43]. Species identification strictly followed the taxonomic standards outlined in “Fauna Sinica” [44,45]. Total length was recorded to the nearest millimeter (1 mm), and wet body weight to the nearest 0.01 g. Fish biomass is expressed using Catch Per Unit Effort (CPUE; the weight of fish per net per hour, in kg/net×h), and fish density is expressed using Density Per Unit Effort (DPUE; the number of fish per net per hour, in ind./net×h). CPUE (kg/h) is calculated by dividing the weight of fish caught in a single net (kg) by the total hours of fishing (h). DPUE (ind./h) is calculated by dividing the number of fish caught in a single net (ind.) by the total hours of fishing (h) [43].

Quantitative water samples and plankton specimens were collected concurrently during fishing operations. At each sampling site, three vertical water layers were sampled: the upper layer (0.5 m below the surface), the middle layer (midway between surface and bottom), and the lower layer (0.5 m above the sediment surface). At each sampling site, in situ monitoring of water temperature (WT), pH, conductivity (Cond), dissolved oxygen (DO), and oxidation-reduction potential (ORP) was conducted using a multiparameter water quality analyzer (YSI ProPlus, Yellow Springs, OH, USA) within the depth range of 0.0 to 0.5 m below the water surface. Transparency was measured in situ with a 20 cm black-and-white Secchi disk [46]. Depth was determined acoustically using a single-beam echosounder synchronized with the Simrad EK80 (Kongsberg Maritime AS (Simrad Fisheries), Horten, Norway) scientific echo sounder during the hydroacoustic fish survey. The detectable depth range fully covers the maximum depth of SXR (80+ m) across all sampling transects. All acoustic depth values were post-calibrated, yielding a final measurement error controlled within ±0.2 m, which meets the accuracy requirement for transect stratification and standardized fish density calculation. Additionally, laboratory analyses were conducted on water samples to determine concentrations of ammonia nitrogen (NH_4_^+^-N), total nitrogen (TN), total phosphorus (TP), and chlorophyll a (Chla), following the analytical methods specified in the Surface Water Environmental Quality Standards (GB 3838-2002) [37,47].

One liter of water was preserved with acetic Lugol’s solution and settled in an Utmohr bottle for 48 h. After sedimentation, the phytoplankton sample was concentrated to 50 mL. The phytoplankton within 0.1 mL subsamples were then enumerated and measured under a 400× magnification using an Olympus microscope (Olympus, Tokyo, Japan). In cases where the phytoplankton cells were colonial or filamentous, an ultrasound crusher (JY88-II; Ningbo, China) was employed to separate them. Phytoplankton were identified following published guidelines [48]. The biomass of phytoplankton species was calculated utilizing the methods described by relevant literature [49].

### 2.3. Hydroacoustic Measurements and Data Processing

The hydroacoustic survey was conducted during daylight in October 2022 and April 2023. A Simrad EK80 scientific echo sounder (equipped with a split-beam transducer, model ES200-7C, operating frequency 200 kHz, half-power beam width (3 dB beam width) 7°) was used for data acquisition [50]. The transducer was mounted on the starboard side of the vessel, positioned one-third of the hull length from the bow, and submerged to a depth of 0.8 m below the water surface. A Garmin GPS 17 × HVS unit recorded the vertical geographical position and hydroacoustic trajectory in real-time, while the echosounder, interfaced with a laptop, displayed and stored data via the software included with the EK80. Water temperature and salinity, essential for data acquisition, were measured using a YSI6600V2 monitor (Yellow Springs, OH, USA). To reduce vessel noise, the survey followed zigzag transects at a speed of 6–8 km·h^−1^ [51]. Starting at the reservoir head and ending at the tail (Figure 1), the route covered the entire reservoir, with the survey repeated twice to achieve a degree of coverage (Aglen’s metric) greater than 6, which is considered adequate for acoustic surveys [50,52].

The hydroacoustic data were converted and post-processed with Sonar5_Pro software V 6.0.2 (Lindem Data Acquisition, Oslo, Norway). Due to dispersed fish signals, blind detection zones and areas with significant interference—specifically, 1 m below the transducer and 0.5 m above the probe bottom—were manually excluded to minimize noise [53,54]. Echograms from all sections were carefully examined and manually edited to reconstruct the bottom surface as needed. Bubble and vessel noise exceeding target signals was removed from echograms [55]. Additionally, the minimum threshold in the target intensity echo image was set to −70 dB to filter out weak scatterer signals like plankton.

This study estimates fish length using the empirical formula for fish target strength (*TS*) versus body length [56]:

(1) TS=20logL−71.9

where *TS* (dB) represents the target strength of the fish, and *L* (cm) denotes the body length of the target fish.

The conversion from total length (*TL*) to fish weight (*W*) follows a power-function model:

(2) $$W=a{TL}^{b}$$

where W is the fish weight and a and b were the species-specific parameters, fitted using length-weight data from sampled catches [57,58]. Biomass per transect was calculated by multiplying mean acoustic-derived fish density by the average weight, inferred from mean target strength (TS). Total biomass for the surveyed area was then computed as a weighted average, incorporating spatial variations in fish distribution and density.

Fish density was calculated using the relevant research methods [59]. The fish density for the survey transect was obtained by dividing the number of individual echoes by the detection volume v, using the following formula:

(3) ρ=n∑i=ki=1vi

where ρ is fish density (individuals/m^3^), n is the number of fish echo signals detected, k is the total number of units in the survey section, and i is the i^(th) unit within the survey section.

### 2.4. Statistical Analyses

Hydroacoustic surveys provided spatially continuous estimates of fish density and target-strength (TS) distribution across the entire reservoir. Concurrent gillnet catches at each sampling site supplied species composition, length-frequency data, and species-specific length–weight relationships. To combine the two datasets, we matched the size classes observed in gillnet catches to corresponding TS ranges using the Foote (1987) [56] TS–L equation (see Section 2.3). This allowed us to infer which size groups (and, by spatial co-occurrence, which taxa) contributed most to acoustic echoes in different zones. Species-level attribution of acoustic targets was therefore based on the spatial co-occurrence of specific size classes in net catches at the same locations, rather than on acoustic signals alone. This catch–acoustic integration approach follows established protocols for reservoir fish surveys [43,50,51].

Fish were grouped by feeding habit: carnivorous, planktivorous, omnivorous, or herbivorous [44,45]. For the horizontal distribution characteristics of fish across different survey areas, spatial distribution modeling was conducted using ArcGIS 10.2 software (Environmental Systems Research Institute (Esri), Redlands, CA, USA). Calculated fish density values for each grid cell and corresponding GPS location data for cell centers were imported into ArcGIS. Raster interpolation was performed using the inverse distance weighting method to generate fish density distribution maps.

Mantel test and Pearson correlation coefficient was employed to determine the relationship between fish spatial distribution and environmental factors. The Mantel test is a statistical method that assesses the correlation between two distance (or dissimilarity) matrices, each representing a different set of variables, thereby enabling direct evaluation of associations between two distinct types of multivariate data. Given the ecological context, where environmental gradients and plankton community composition are inherently multivariate and interdependent, we applied the Mantel test to examine the relationship between fish abundance and key ecological variables. All analyses were conducted in R Studio v4.0.3 using the “corrplot” package for Pearson correlation, the “vegan” package for Mantel testing and the “tidyverse” suite for data wrangling and visualization. All analyses were performed using R Studio v4.0.3 and ArcGIS 10.2 software [60].

## 3. Results

### 3.1. Seasonal Dynamics of Fish Assemblages and Characteristics

In October 2022 (autumn), a total of 21 fish species (674 individuals) were recorded, belonging to 4 orders, 6 families and 15 genera. In April 2023 (spring), 22 species and 1839 individuals were documented (Table 1). The assemblage comprised 4 orders, 8 families, and 17 genera, with 16 species shared across seasons. Sixteen species were common to both seasons. Cyprinidae was the dominant family in both seasons, accounting for 71.4% of species in autumn and 68.2% in spring.

**Figure 2:**
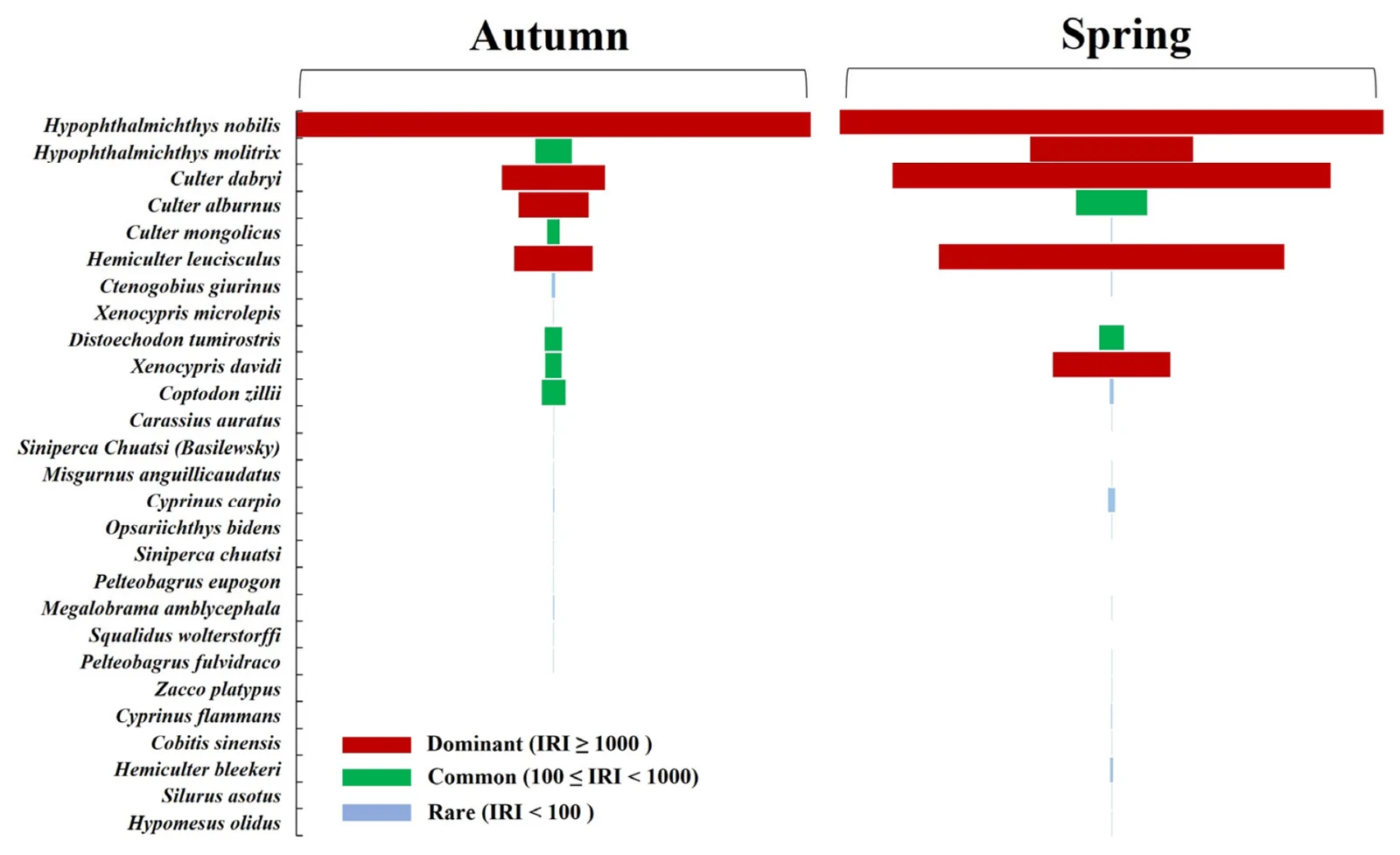
Seasonal shifts in the Index of Relative Importance (IRI) of all recorded fish species in Shanxi Reservoir 2022–2023. The calculation of IRI integrates relative abundance, relative biomass and occurrence frequency data collected via 12 h gillnet deployments across 18 sampling sites in both seasons; longer bar length corresponds to higher relative importance of the target species.

The Index of Relative Importance (IRI) was used to classify species: IRI ≥ 1000 as dominant, 100 ≤ IRI < 1000 as common, and IRI < 100 as rare. Dominant species shifted markedly between seasons (Table 1). In autumn, the dominant species were *H. nobilis* (IRI = 9544), *C. dabryi* (IRI = 1919), *H. leucisculus* (IRI = 1468), and *C. alburnus* (IRI = 1313). Common species included *H. molitrix*, *C. zillii*, *D. tumirostris*, *X. davidi*, and *C. mongolicus*; all other species were rare (Figure 2). In spring, the dominant species were *H. nobilis*, *C. dabryi*, *H. leucisculus*, *H. molitrix*, and *X. davidi*; *C. alburnus* and *D. tumirostris* were common; the remaining species were rare (Figure 2).

**Figure 3:**
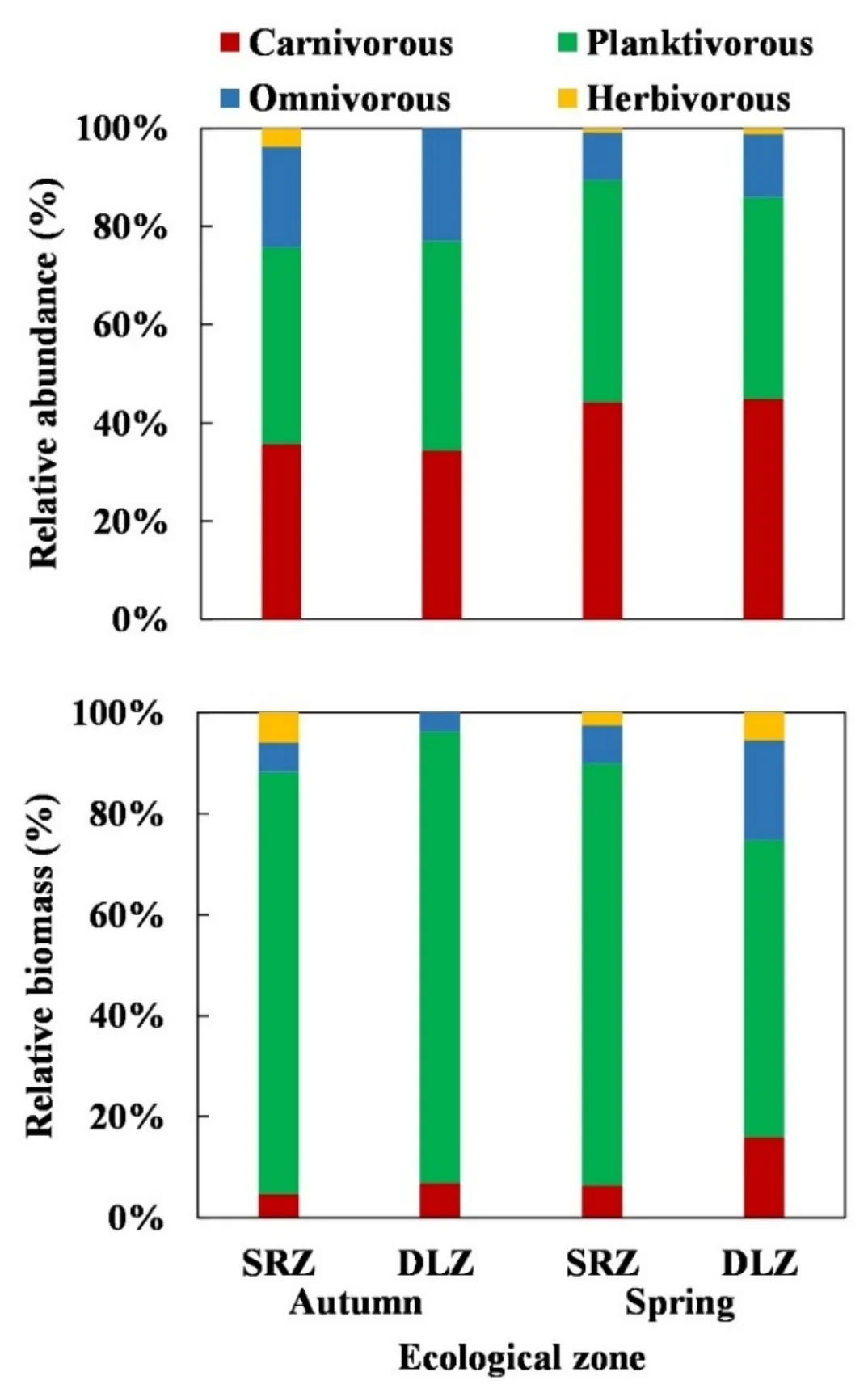
Seasonal and zonal variation in the proportional abundance of four trophic guilds (carnivorous, planktivorous, omnivorous, and herbivorous fish) in Shanxi Reservoir, shown separately for the shallow riverine zone (SRZ) and deep lake zone (DLZ) across autumn (October 2022) and spring (April 2023) surveys.

Based on feeding habits, fish were classified into four trophic guilds: carnivorous, planktivorous, omnivorous, and herbivorous. Seasonal shifts were most pronounced in the relative abundances of planktivorous, carnivorous, herbivorous, and omnivorous fish (Figure 3). In the SRZ, planktivorous species (e.g., *H. nobilis*, *H. molitrix*, and *H. leucisculus*) decreased from 45.2% in spring to 39.9% in autumn. In contrast, in the DLZ their proportion remained similar (spring 41.1%, autumn 42.6%). Carnivorous fish (e.g., *C. alburnus* and *C. dabryi*) declined markedly from spring to autumn in both SRZ (44.3% → 35.8%) and DLZ (44.9% → 34.5%). Regarding omnivorous fish, represented by *Xenocypris microlepis*, *Xenocypris davidi*, and *C. zillii*, their proportion in the SRZ rises markedly from spring (9.7%) to autumn (20.4%). Correspondingly, the DLZ also witnesses an increase in the proportion of omnivorous fish in autumn (22.9%) as opposed to spring (12.9%).

Through integrated acoustic in situ surveys and fish catch data, we found that fish assemblage in SXR are predominantly composed of small- to medium-sized individuals, exhibiting a markedly non-uniform horizontal distribution. Mean acoustic density was 2.67 ± 2.55 ind/1000 m^3^ in autumn and declined to 0.89 ± 0.68 ind/1000 m^3^ in spring (Table 2). Notably, these acoustic densities are not directly comparable to raw catch counts (674 individuals in autumn vs. 1839 in spring), owing to fundamental differences between the two methods: (i) units (volumetric density vs. discrete sample-based counts), (ii) spatial coverage (integrated across the entire water column vs. limited to discrete benthic sampling sites), and (iii) size selectivity (acoustics detect fish across the full size spectrum, whereas gillnets selectively capture individuals within a narrow, gear-specific size range). Consequently, we treat acoustic and catch data as complementary—rather than interchangeable—sources of information: acoustic data primarily reveal the spatial distribution patterns of the numerically dominant small-size fraction, while gillnet catches provide insights into species composition and key biological traits (e.g., length, maturity, age) of the commercially or ecologically relevant, catchable size classes.

### 3.2. Spatial Distribution Patterns of Fish Assemblages

**Figure 4:**
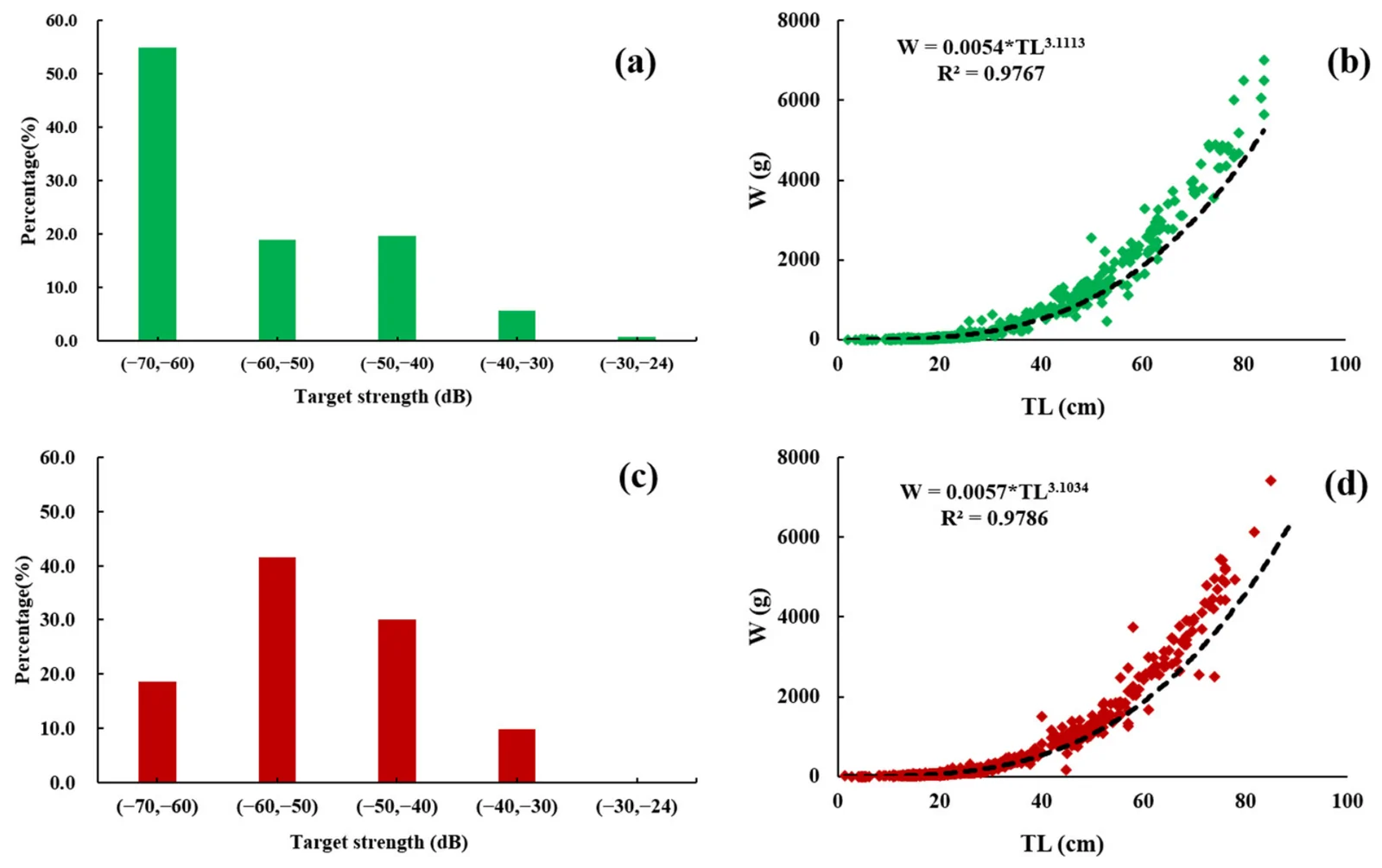
Hydroacoustic target strength (TS) frequency distributions and empirically fitted length −weight relationships for fish assemblages in Shanxi Reservoir (SXR), shown for autumn (panels (**a**,**b**)) and spring (panels (**c**,**d**)). Both seasons were numerically dominated by small − to medium −sized individuals, as indicated by the TS distributions and corresponding length estimates.

The autumn acoustic survey recorded 25,894 fish signals, with a mean target strength (TS) of −57.98 ± 10.46 dB, dominated by the −70 to −60 dB range. Using the TS-to-length conversion formula, the estimated mean fish length was 4.97 cm. The length-weight relationship fitted from autumn catch samples were *W* = 0.0054 × *TL*^3.1114^ (R^2^ = 0.9767) (Figure 4a,b). In spring, 16,115 signals were recorded, mean TS = −51.65 ± 8.16 dB, dominated by −60 to −50 dB, corresponding to a mean length of 10.28 cm. The spring length-weight relationship was *W* = 0.0057 × *TL*^3.1034^ (R^2^ = 0.9786) (Figure 4c,d).

**Figure 5:**
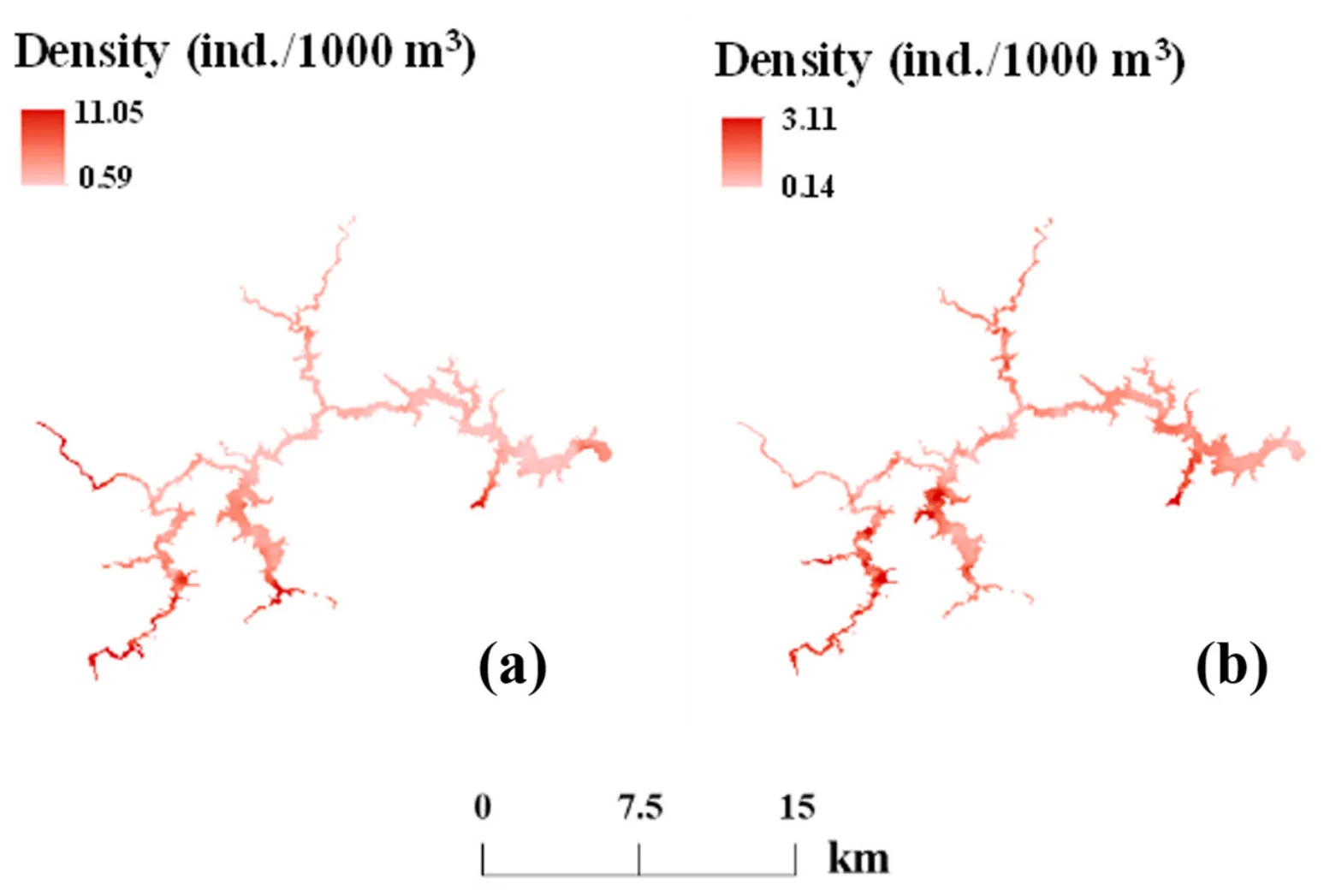
IDW-interpolated horizontal distribution maps of hydroacoustic fish density (ind/1000 m^3^) across Shanxi Reservoir (SXR) for autumn (panel (**a**)) and spring (panel (**b**)). Season-specific color scales are used because density ranges differ substantially between seasons (autumn: 0.59–11.05 ind/1000 m^3^; spring: 0.14–3.3.11 ind/1000 m^3^); a unified scale would obscure fine-scale spatial patterns within each season. Within each panel, color intensity (light to dark) corresponds to increasing relative density.

Fish were unevenly distributed across the reservoir. Overall mean acoustic density across both seasons was 2.67 ± 2.55 ind/1000 m^3^ (95% CI: 2.20–3.14), with autumn density ranging from 0.59 to 11.06 ind/1000 m^3^ (Table 2). In both autumn and spring, fish concentrated in the shallow riverine zone, with small-sized species (*C. alburnus*, *C. dabryi*, and *H. leucisculus*) being the most abundant. Aggregations of small-sized *C. dabryi* and *H. leucisculus* were also found at some inlets of the deep lake zone (Figure 5). Note that overall hydroacoustic fish density was markedly higher in autumn (mean: 2.67 ind/1000 m^3^) than in spring (mean: 0.89 ind/1000 m^3^); see Table 2 for detailed seasonal comparisons.

Similarly, the acoustic mean lengths (4.97 cm in autumn, 10.28 cm in spring) appear smaller than the sizes dominating gillnet catches (*H. nobilis* and *Culter* spp.), because acoustics numerically count the huge number of small individuals (e.g., *H. leucisculus*) that escape gillnet meshes, whereas large fish, though biomass dominant, are far fewer in number and contribute little to density estimates. Although the absolute mean lengths derived from hydroacoustics and net catches differed slightly (which is expected given the size-selectivity of gillnets), both methods consistently indicated that small- to medium-sized fish constituted the majority of the population in both seasons.

### 3.3. Association Between Fish Assemblage Distribution and Environmental Factors

**Figure 6:**
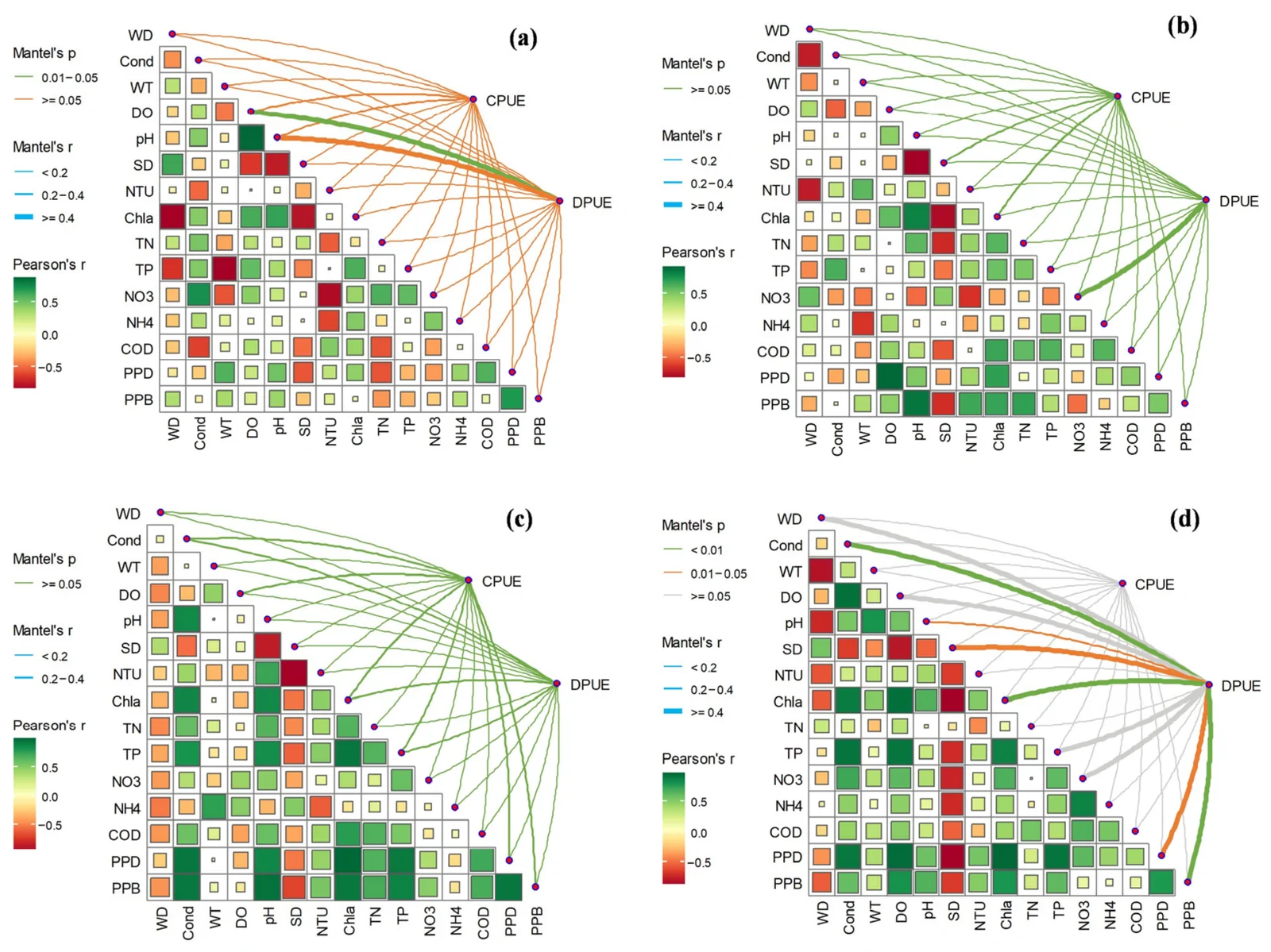
Associations between fish density (DPUE), fish biomass (CPUE), and environmental variables evaluated via Mantel tests and Bonferroni −corrected Pearson correlation analyses, presented for four season-zone combinations: (**a**) shallow riverine zone in autumn (SRZ-Autumn), (**b**) deep lake zone in autumn (DLZ-Autumn), (**c**) shallow riverine zone in spring (SRZ-Spring), and (**d**) deep lake zone in spring (DLZ-Spring). All analyses were implemented in R using the “vegan” packages (Mantel test, 9999 permutations, Bray–Curtis dissimilarity for fish assemblage, Euclidean distance for environmental variables) and “corrplot” (Pearson correlation) packages. Abbreviations: DPUE, fish density per unit effort; CPUE, fish biomass per unit effort; WD, water depth; Cond, conductivity; WT, water temperature; DO, dissolved oxygen; pH, pH value; SD, Secchi disk depth; NTU, turbidity; Chla, chlorophyll a; TN, total nitrogen; TP, total phosphorus; NO3, nitrate nitrogen; NH4, ammonia nitrogen; COD, chemical oxygen demand; PPD, phytoplankton density; PPB, phytoplankton biomass. The heatmap uses color to represent the magnitude and direction of Pearson correlation coefficients (r): green indicates positive correlation, red indicates negative correlation, and darker colors denote stronger correlations. The legend above the heatmap displays *p*-values from the Mantel test: green indicates *p*-values between 0.01 and 0.05; orange indicates *p* ≥ 0.05. Grey lines in the Mantel section indicate non-significant associations (*p* ≥ 0.05).

Mantel tests and Pearson correlation analyses revealed season- and zone-specific relationships between multiple environmental variables and fish density (DPUE) or biomass (CPUE) (Figure 6). In the SRZ during autumn, phytoplankton density correlated positively with water temperature, pH and COD, and negatively with Secchi depth (SD). Fish assemblage density was significantly associated with dissolved oxygen (DO; *p* < 0.05) (Figure 6a). In the DLZ during autumn, overall environmental correlations were weak and non-significant for fish metrics (*p* > 0.05), but phytoplankton biomass correlated positively with pH and turbidity (NTU) and negatively with SD (r ≈ −0.5). Fish density again showed a significant link to DO (*p* < 0.05) (Figure 6b). In the SRZ during spring, no significant correlations were detected between fish density/biomass and environmental variables (*p* > 0.05); however, phytoplankton density/biomass exhibited robust positive correlations with conductivity (Cond), pH, NTU, TN, TP, Chla and COD, and negatively with SD (r ≈ −0.5) (Figure 6c). In the DLZ during spring, fish density was highly significant correlated with Cond, chlorophyll a (Chla) and phytoplankton biomass (PPB) (*p* < 0.01), and significantly correlated with SD and phytoplankton density (PPD) (*p* < 0.05). Phytoplankton variables also showed significant positive correlations with Cond, DO, pH, NTU and TP, and negatively correlations with SD (r ≈ −0.5, *p* < 0.05) (Figure 6d).

## 4. Discussion

This exploratory two-season study describes the seasonal and spatial distribution patterns of fish assemblages in a subtropical river-type reservoir (Shanxi Reservoir, SXR) and examines associations with environmental variables. The fish community exhibits marked seasonal succession, with dominant taxa including *H. nobilis*, *C. dabryi*, and *H. leucisculus*. Planktivorous and carnivorous fish were proportionally more abundant in spring, while omnivorous fish increased in autumn. Fish were predominantly concentrated in the shallow riverine zone (SRZ), and the assemblage was dominated by small- to medium-sized individuals. Dissolved oxygen was associated with fish distribution patterns in autumn, while in spring, fish density in the deep lake zone (DLZ) showed significant correlations with conductivity, Chla, and phytoplankton density and biomass. As the first systematic survey of fish assemblages in SXR, this study establishes a baseline for future long-term monitoring and offers preliminary observations that may inform future water quality, ecological restoration, and fisheries management planning in subtropical river-type reservoirs.

While our sampling was restricted to two seasons within a single year, precluding robust assessments of interannual variability or persistent successional trends, the observed patterns nonetheless provide a valuable baseline for future long-term monitoring efforts in this under-studied reservoir system. Methodologically, we treated hydroacoustics and gillnetting as complementary rather than redundant approaches: the former furnished continuous, non-destructive density profiles across the full water column and all size cohorts, critical for mapping horizontal distributions, whereas the latter, though constrained by mesh selectivity and limited spatial reach, delivered species-level taxonomic and biological data (e.g., length–weight and trophic attributes) essential for calibrating acoustic returns. Acknowledging that neither method independently captures the full assemblage, acoustics cannot discriminate taxa, while nets under sample certain size fractions, the observed quantitative mismatches were anticipated and do not compromise our core inferences, as each technique compensates for the other’s blind spots. We further recognize that employing a generic TS–L equation across a morphologically diverse cyprinid fauna, conducting daytime surveys susceptible to diel vertical shifts, and inferring species identities indirectly from catch-to-echo alignment may introduce moderate biases. Nevertheless, the overall congruence between acoustically derived spatial patterns and catch-based compositional data reinforces the robustness of our primary conclusions regarding seasonal and spatial community dynamics.

A potential concern is the apparent discrepancy between acoustic densities estimates and net-catch abundances: acoustic surveys recorded higher fish density in autumn (2.67 ind/1000 m^3^) than in spring (0.89), whereas gillnet catches yielded substantially more individuals in spring (1839) than in autumn (674). This contrast does not reflect methodological error, but rather stems from fundamental differences in what each method measures. Acoustic density represents a volumetric, size-integrated estimate across the entire water column, while net catches provide a gear-selective, spatially discrete count—primarily from benthic or near-bottom habitats. Spring coincides with the spawning season for many species, prompting aggregations in shallow, inshore areas where gillnets are most effective. This behavioral concentration increases catchability without necessarily elevating overall volumetric density throughout the reservoir. Similarly, the acoustic mean lengths (4.97 cm in autumn, 10.28 cm in spring) appear smaller than the sizes dominating gillnet catches (*H*. *nobilis* and *Culter* spp.), because acoustics numerically count the huge number of small individuals (e.g., *H*. *leucisculus*) that escape gillnet meshes, whereas large fish, though biomass-dominant, are far fewer in number and contribute little to density estimates. Thus, rather than contradicting each other, the two methods sample different components of the same assemblage. This synergistic interpretation aligns with well-established best practices in reservoir fisheries research, where integrated use of hydroacoustics and active/passive gear-based sampling is widely recommended [43,50,51].

No prior studies have documented the fish community structure of SXR. In this study, we documented 27 fish species, fewer than historical records (CJ. Yin Unpublished data). Cypriniformes dominated the assemblage, accounting for 71.4% of all species, consistent with typical inland reservoir communities. The community was largely composed of artificially stocked and small-sized species, particularly *H. nobilis*, *H. molitrix*, and *H. leucisculus*. The dominance of the first two reflects their intentional stocking for water conservation-oriented fisheries, while *H. leucisculus* is a naturally abundant, small-sized species commonly dominant in inland waters. Notably, dominant species exhibit pronounced seasonal succession: *H. nobilis*, *C. dabryi*, and *H. leucisculus* were dominant in both seasons, whereas *H. molitrix* increased substantially in spring. These temporal shifts likely reflect adapting foraging strategies driven by seasonal temperature fluctuations and hydrological pulse events [61,62,63].

The observed seasonal shift in trophic guilds (e.g., planktivores and carnivores) peaking in spring, while omnivores increasing in autumn, which has direct practical implications for fisheries and ecosystem management. Spring, characterized by higher phytoplankton biomass (as indicated by strong correlations with Chla), which hypothetically could represent a more favorable period for stocking filter feeding species (*H. nobilis*, *H. molitrix*) if algal control is a management objective [35]. Conversely, autumn stocking of omnivores may warrant further evaluation to assess potential competition with native species. These tentative implications should be tested through dedicated stocking experiments and long-term monitoring before being adopted as management prescriptions.

Comparative analysis of feeding habits is crucial for understanding aquatic community structure [64]. In both autumn and spring, planktivorous and carnivorous fish constituted a high proportion of total abundance; however, planktivores dominated in terms of biomass, indicating not only abundant planktonic prey but also a well-developed representation of higher trophic levels. Omnivorous fish showed higher abundance in autumn and a marked increase in biomass proportion in the DLZ during spring. Compared to the relatively stable seasonal dynamics reported for large reservoirs such as the Three Gorges Reservoir [65], the shorter impoundment cycle and frequent monsoon-induced flow fluctuations in SXR [33] likely contribute to the pronounced changes in trophic composition. These spatiotemporal variations are further interpreted as adaptive responses to pulsed nutrient inputs characteristic of subtropical riverine ecosystems [66,67].

Spatial interpolation analysis confirmed that fish were predominantly distributed in the SRZ, with small-bodied species exhibited greater abundance in the SRZ, this pattern consistent with findings from numerous riverine reservoir studies [68,69,70]. For instance, floodplains and shallow-water habitats in the Paraná River basin function as critical spawning and foraging grounds [23]. The presence of small-sized *Culter* spp. and *H. leucisculus* at DLZ inlets further supports the role of transitional zones as ecological corridors, facilitating habitat connectivity between SRZ and DLZ [19]. Moreover, the lower fish density and diminished environmental associations observed in the DLZ during autumn indicate that deepwater habitats primarily serve as thermal refuges rather than primary feeding or spawning grounds, particularly under conditions of pronounced surface water temperature variability [14]. Consequently, the influence of environmental drivers on fish distribution was substantially attenuated in the DLZ.

Our results indicate that DO and Chla are significantly associated with fish assemblage variability, suggesting their potential utility as candidate indicators for future monitoring programs. However, whether specific thresholds (e.g., DO concentrations or Chla levels) could serve as early-warning signals for ecosystem degradation or management intervention remains an open hypothesis. Testing this would require rigorous, multi-year observational monitoring coupled with targeted experimental studies to establish causal linkages and quantify response lags. Future research should therefore investigate whether sustained deviations in DO or Chla consistently precede detectable shifts in fish assemblage structure or function, and whether such metrics can be translated into actionable, adaptive management frameworks.

*Mantel tests* and Pearson correlation analyses revealed that DO consistently limited fish distribution across both seasons and spatial zones, a pattern consistent with findings from North American river-type reservoirs, where oxygen availability has been identified as a critical determinant of fish habitat selection [32]. During spring in the DLZ, fish density showed a significant positive correlation with Chla concentration, underscoring the foundational role of phytoplankton biomass in aquatic food webs and its directly influence on the spatial aggregation of planktivorous and omnivorous fish [7]. Notably, SD showed a consistent negative correlation with phytoplankton density across all seasons, suggesting that turbidity, driven by both external factors (e.g., monsoon-induced runoff and reservoir operational regimes) and internal feedbacks (e.g., self-shading by dense phytoplankton blooms), may indirectly shape fish assemblage structure by attenuating light penetration and impairing visual prey detection for planktivorous species [20].

The observed associations between fish assemblage patterns and hydrological or nutrient variables align with the expectation that reservoir ecosystems are shaped by both natural processes and human-induced. As preliminary, hypothesis-generating insights for future management, we propose the following: (i) preserving natural shoreline complexity and submerged macrophytes in the SRZ may support critical spawning and nursery habitats, though this potential benefit requires empirical validation; (ii) Targeted control of nutrient inputs from upstream tributaries, particularly during the pre-flood season, could help mitigate eutrophication- driven shifts in trophic structure and guild composition; and (iii) Engaging local fishers in citizen-science monitoring (e.g., recording catch composition and water transparency) could supplement formal surveys and support long-term tracking of ecological change. These recommendations are currently speculative and should be rigorously tested through focused, adaptive research before being adopted as formal management actions.

Previous investigations, along with reports from local fishermen, indicate that anthropogenic hydrological regulation, particularly flood control and water supply operations, has altered the natural hydrological regime of the SXR. This disruption has been linked to declines in catches of fish that thrive in turbulent waters within local fisheries. These changes likely affect fish habitats, posing risks to certain fish populations [9]. To support both ecological preservation and fisheries sustainability, it is important for the SXR to maintain reservoir storage capacity that safeguards shallow spawning habitats. Moreover, long-term monitoring of fish communities using hydroacoustic technology is advised to observe how these communities respond to management actions. This approach has proven effective in adaptive management of subtropical reservoir ecosystems [43,50]. Beyond its application in the SXR, combining hydroacoustic surveys with simultaneous gillnet sampling offers a replicable and cost-efficient method for broader monitoring efforts in similar river-type reservoirs. Hydroacoustics provide spatially continuous, non-invasive estimates of fish density across space, while net sampling delivers species-specific biological information such as size, weight, and diet composition. By integrating these two methods, managers can calibrate acoustic data with local catch information, enabling comparisons across different systems and early detection of changes in fish communities. We recommend adopting this combined approach in regional monitoring programs, especially where resources for extensive traditional surveys are limited. Future research should extend monitoring to include these extreme hydrological events and incorporate stable isotope analysis to better understand food web interactions within the fish communities [16]. Additionally, studying the ecological effects of invasive species like *C. zillii* on native fish populations would enhance understanding of the SXR’s ecosystem dynamics, as well as support informed conservation and management decisions.

## 5. Conclusions

As an exploratory two-season baseline study, this work describes the spatiotemporal patterns of fish assemblages and their associations with environmental variables in SXR, a subtropical river-type reservoir, using an integrated multi-method survey approach. The principal preliminary findings are summarized as follows: (1) fish assemblages in SXR display pronounced seasonal and spatial heterogeneity. Cyprinidae constitutes the dominant family, with 21 species recorded in autumn and 22 in spring, accompanied by marked seasonal turnover in dominant taxa (e.g., the persistent dominance of *H. nobilis* and the elevated proportional importance of *H. molitrix* during spring). Planktivorous and carnivorous fish are proportionally more abundant in spring, while omnivorous fish increase in autumn, patterns that may reflect responses to seasonal shifts in resource availability and environmental conditions. (2) Spatially, hydroacoustic fish density is concentrated in the SRZ, where small-to-medium-sized species form the bulk of the assemblage. Deep lake inlets appear to function as transitional zones facilitating fish movement, while the DLZ is characterized by comparatively low fish density and may serve as a thermal refuge. (3) Within the DLZ, fish assemblage patterns show seasonal variability that is statistically associated with environmental parameters, including dissolved oxygen, conductivity, Chla, and phytoplankton density and biomass. Collectively, this work establishes the first systematic baseline for fish communities in SXR and provides a reference case for subtropical river-type reservoir monitoring. The correlational findings generate hypotheses for future testing and may inform preliminary adaptive fisheries management strategies, pending further validation. Future investigations should expand temporal coverage across multiple years and seasons, incorporate stable isotope analysis to examine trophic interactions, and evaluate the ecological impacts of non-native species.

## Figures and Tables

**Table 1:** Full inventory of fish species recorded in Shanxi Reservoir across two sampling campaigns (October 2022 autumn post-flood period, April 2023 spring spawning period). IRI classification follows the standardized rule: ≥1000 = dominant, 100 ≤ IRI < 1000 = common, <100 = rare.

Order	Family	Genus	Species Name	Common Name	Code	Historical Data	October 2022	April 2023	Native/Non-Native
Cypriniformes	Cyprinidae	*Zacco*	*Zacco platypus*	Pale chub	ZPL	+		+	Native
		*Opsariichthys*	*Opsariichthys bidens*	Chinese hooksnout carp	OBI	+	+	+	Native
		*Ctenopharyngodon*	*Ctenopharyngodon idellus*	Grass carp	CID	+			Native
		*Mylopharyngodon*	*Mylopharyngodon piceus*	Black carp	MPI	+			Native
		*Distoechodon*	*Distoechodon tumirostris*	Distocodon carp	DTU	+	+ (∮)	+ (∮)	Native
		*Xenocypris*	*Xenocypris davidi*	David’s xenocypriid	XDA	+	+ (∮)	+ (*)	Native
			*Xenocypris microlepis*	Yellowfin	XMI		+		Native
		*Hypophthalmichthys*	*Hypophthalmichthys molitrix*	Silver carp	HMO	+	+ (∮)	+ (*)	Native
			*Hypophthalmichthys nobilis*	Bighead carp	HNO	+	+ (*)	+ (*)	Native
		*Hemiculter*	*Hemiculter leucisculus*	Sharpbelly	HLE	+	+ (*)	+ (*)	Native
			*Hemiculter bleekeri*	Bleeker’s sharpbelly	HBL	+		+	Native
		*Culter*	*Culter mongolicus*	Mongolian culter	CMO	+	+ (∮)	+	Native
			*Culter alburnus*	Topmouth culter	CAL	+	+ (*)	+ (∮)	Native
			*Culter dabryi*	Dabry’s culter	CDA	+	+ (*)	+ (*)	Native
			*Culter erythropterus*	Redfin culter	CER	+			Native
		*Megalobrama*	*Megalobrama amblycephala*	Wuchang bream	MAM	+	+	+	Native
		*Hemibarbus*	*Hemibarbus maculatus*	Spotted steed	HMA	+			Native
		*Squalidus*	*Squalidus wolterstorffi*	Wolterstorff’s gudgeon	SWO		+		Native
		*Pseudorasbora*	*Pseudorasbora parva*	Stone moroko	PPA	+			Native
		*Abbottina*	*Abbottina rivularis*	Chinese false gudgeon	ARI	+			Native
		*Spinibarbus*	*Spinibarbus denticulatus*	Denticulate spinibarbus	SDE	+			Native
		*Acrossocheilus*	*Acrossocheilus wenchowensis*	Wenchuan barbel	AWE	+			Native
		*Cyprinus*	*Cyprinus carpio*	Common carp	CCA	+	+	+	Native
			*Cyprinus flammans*	Red common carp	CFL	+		+	Native
		*Carassius*	*Carassius auratus*	Crucian carp	CAU	+	+	+	Native
	Cobitidae	*Misgurnus*	*Misgurnus anguillicaudatus*	Oriental weatherfish	MAN	+	+	+	Native
		*Cobitis*	*Cobitis sinensis*	Chinese spined loach	CSI	+			Native
Siluriformes	Bagridae	*Pelteobagrus*	*Pelteobagrus fulvidraco*	Yellowhead catfish	PFU	+	+		Native
			*Pelteobagrus eupogon*	Mandarin Chinese	PEU	+	+		Native
		*Pseudobagrus*	*Pseudobagrus ussuriensis*	Bagrid catfish	PSP	+		+	Native
	Siluridae	*Silurus*	*Silurus asotus*	Amur catfish	SAS			+	Native
Salmoniformes	Osmeridae	*Hypomesus*	*Hypomesus olidus*	Pond smelt	HOL	+		+	Native
Anguilliformes	Anguillidae	*Anguilla*	*Anguilla marmorata*	Giant mottled eel	AMA	+			Native
Perciformes	Cichlidae	*Coptodon*	*Coptodon zillii*	Redbelly tilapia	CZI	+	+ (∮)	+	Non-native
	Gobiidae	*Ctenogobius*	*Ctenogobius giurinus*	Barcheek goby	CGI	+	+	+	Native
		*Rhinogobius*	*Rhinogobius cliffordpopei*	Clifford Pope’s goby	RCL	+			Native
	Taenioididae	*Odontamblyopus*	*Odontamblyopus rubicundus*	Dark sleeper	ORU	+			Native
	Channidae	*Channa*	*Channa argus*	Northern snakehead	CAR	+			Native
	Odontobutidae	*Odontobutis*	*Odontobutis obscurus*	Dark sleeper	OOB	+			Native
	Sinipercidae	*Siniperca*	*Siniperca chuatsi*	Aucha Perch	SCH	+	+	+	Native
			*Siniperca Chuatsi* (Basilewsky, 1855)	Mandarin fish	SCB		+		Native

Note: “+” indicates the species was detected in that season. Symbols in parentheses indicate the relative importance category based on the Index of Relative Importance (IRI): (*) = dominant species (IRI ≥ 1000); (∮) = common species (100 ≤ IRI < 1000); a plain “+” (without symbols) indicates the species was detected but is considered rare (IRI < 100). All species names are presented as standard binomial Latin names following the journal’s style.

**Table 2:** Seasonal comparison of hydroacoustic fish density (ind/1000 m^3^) partitioned by estimated body length groups in Shanxi Reservoir (SXR). Values are presented separately for autumn (October 2022) and spring (April 2023) surveys to illustrate seasonal differences in size-structured density across the reservoir.

Group	Mean (Ind/1000 m^3^)		Range (Ind/1000 m^3^)		95% Confidence Interval	
	Mean	SD	Maximum	Minimum	Lower Limit	Upper Limit
Reservoir (Autumn)	2.67	2.55	11.06	0.59	2.20	3.14
1.24~3.94	1.29	1.40	6.95	0.22	1.03	1.55
3.94~12.45	0.45	0.56	4.17	0.00	0.35	0.55
12.45~39.36	0.62	0.88	5.34	0.00	0.46	0.78
39.36~124.45	0.13	0.16	0.99	0.00	0.11	0.16
≥124.45	0.02	0.04	0.29	0.00	0.01	0.03
Reservoir (Spring)	0.89	0.68	3.60	0.14	0.78	1.01
1.24–3.94	0.16	0.17	1.32	0.00	0.13	0.19
3.94–12.45	0.33	0.29	1.51	0.03	0.29	0.38
12.45–39.36	0.30	0.28	1.55	0.01	0.25	0.34
39.36–124.45	0.10	0.13	0.81	0.00	0.08	0.12
≥124.45	0.00	0.00	0.00	0.00	0.00	0.00

## Data Availability

The original contributions presented in the study are included in the article, further inquiries can be directed to the corresponding authors.
